# Options and Considerations in the Management of Peritoneal Disease in Patients with Small Bowel Neuroendocrine Tumors

**DOI:** 10.1007/s11864-025-01364-y

**Published:** 2025-10-13

**Authors:** Jeremy Chang, Udhayvir S. Grewal, Scott K. Sherman, James R. Howe

**Affiliations:** 1 Department of Surgery, Division of Surgical Oncology and Endocrine Surgery, University of Iowa Carver College of Medicine, Iowa City, IA USA; 2 Winship Cancer Institute of Emory University, Atlanta, GA USA

**Keywords:** Peritoneal metastases, Small bowel neuroendocrine tumor, Cytoreductive surgery, Somatostatin analogs, Peptide receptor radionuclide therapy

## Abstract

Peritoneal metastases (PM) in small bowel neuroendocrine tumors (SBNET) are challenging. These patients have worse oncologic outcomes and may have symptoms related to mechanical obstruction and hormone production. Difficult decisions apply in diagnosis, surgical selection, postoperative systemic therapy, and surveillance. To aid in these decisions, we routinely recommend obtaining somatostatin receptor based functional imaging (i.e. DOTA PET/CT) and arterial and venous phase CT preoperatively to evaluate disease burden and guide surgical planning. Disease biology should also guide surgical management. The presence of synchronous liver metastases should not exclude patients from surgery. For patients with PM and grade 1 or 2 well differentiated SBNETs, we recommend aggressive surgical cytoreduction with the goal of a completeness of cytoreduction (CC) of 0 or 1 and > 70% cytoreduction of liver metastases. For high grade (G3) well differentiated SBNETs, surgical intervention may still be considered. In patients where the extent of disease does not allow for effective cytoreduction, or where patient comorbidities preclude extensive surgery, palliative surgeries or interventions may be preferred. Postoperatively, radiologic surveillance is important to evaluate for disease progression. Some SBNET patients presenting without PM are at risk of developing PM in follow-up, especially those with liver metastases or high T stage. In patients with progression or inoperable disease, systemic therapy including somatostatin analogs (SSAs), chemotherapy or peptide receptor radionuclide therapy (PRRT) may be potential options, although the latter may pose increased risk of bowel obstruction. When cytoreducton and systemic therapy are no longer options, palliative measures should be employed. Because of this complexity, management of PM in SBNET patients is a multidisciplinary collaborative effort.

## Introduction

Neuroendocrine tumors (NETs) are the most common primary neoplasms of the small intestine. Small bowel NETs (SBNETs) arise from neuroendocrine cells of the digestive tract, known as enterochromaffin cells, which are responsible for hormone secretion during digestion. Their biology usually demonstrates slow growth and favorable long-term survival, however significant clinical and pathological heterogeneity exists. Despite their indolent disease course and non-specific symptomatology, >50% of patients present with metastatic disease [1]. Typically, lymph nodes are the most common site of metastases followed by the liver and the peritoneum [2]. The incidence of peritoneal metastases (PM) in SBNET patients has been reported at approximately 10–30% [3–6]. These are rarely an isolated event among patients with SBNETs, as most will also have lymph node and liver metastases.

Presence of PM has significant implications for oncologic outcomes and quality of life. Peritoneal implants can cause compression or twisting of the bowel, leading to abdominal pain and bowel obstruction. The burden of NET disease correlates with neuroendocrine hormone/biomarker levels, and high-volume peritoneal disease may therefore lead to extensive hormones and bioactive amine production. Hormone oversecretion by neuroendocrine cells can cause symptoms of carcinoid syndrome, including diarrhea, flushing, palpitations, and wheezing [7]. Given the indolent nature of the disease course and potential for long-term survival in SBNET patients, it is important to consider symptom control in treatment plans.

The first guidelines on PM in SBNET were published by the European Neuroendocrine Tumor Society (ENETS) in 2007 [8]. They concluded that data regarding PM management were scarce due to the rarity of the disease and that further studies were necessary to provide evidence-based guidelines. They suggested that knowledge gaps for the optimal method of diagnosis, surgical management, and role for additional treatments remain. This review provides a contemporary update of the literature on PM in SBNETs, focusing on epidemiology, disease pathophysiology, diagnosis, and management options.

## Pathophysiology and Incidence of PM in SBNET

Unlike metastases to other locations, which form through hematogenous (i.e. liver metastases) or lymphatic spread, PM in SBNET are thought to occur through direct spread as the primary tumor grows beyond the serosal layer of the bowel. Supporting this, higher T stage and obstructing tumors are risk factors for PM [9]. For the invading cancer to seed and propagate in the peritoneum, close interaction between the peritoneal microenvironment and the tumor cells is required. The visceral and parietal peritoneum are comprised of three layers together with a fluid film. The fluid film, which sits on top of the mesothelial cells of the peritoneum, functions to maintain hydration and homeostasis of the surrounding tissues and is made up of proteoglycans and glycosaminoglycans, the most prominent of which is hyaluronic acid (HA) [10]. In gastrointestinal cancers, the mechanism of primary tumor spread to the peritoneum is thought to be multifactorial, through regulation of intercellular adhesion molecules, cytokines and various growth factors [11]. In colorectal and ovarian cancer, downregulation of E-cadherin in tumor cells has been found to be associated with a motile tumor biology and increased invasive potential [12, 13]. Adhesion molecules on peritoneal mesothelial cells can interact with receptors, such as CD44, on tumor cells, resulting in cytokine and Tumor Necrosis Factor alpha (TNFa) release, facilitating tumor cell attachment and invasion into the peritoneum [14, 15]. HA in the peritoneal glycocalyx can serve as a binding site for tumor cells through CD44, CD168 and ICAM-1 [16]. Lastly, upregulation of various growth factors, including vascular endothelial growth factor (VEGF), has been demonstrated to be required for the peritoneal metastases’ growth [17]. 

The incidence of PM in SBNET patients reported in the literature varies based upon the series (Table 1). Retrospective studies from tertiary care institutions have reported an incidence of roughly 30% [4, 5]. These may overestimate the true incidence, as these centers generally care for patients with more advanced disease. A study of the Surveillance, Epidemiology, and End Results (SEER) Program of the National Cancer Institute (NCI) database analyzing 13,715 tumors over a 50 year period found that for midgut neuroendocrine tumors the incidence of PM was 13.6% [18]. Similarly, more recent data from the Netherlands Cancer Registry and a multicenter Spanish study found an incidence of PM in SBNET patients to be roughly 13% [3, 6]. In a study of 4114 gastroenteropancreatic neuroendocrine tumors (GEPNETs), PM is most frequently observed in small intestine and colon primary NETs (both at 13% incidence) and less so in appendix, gastric, and pancreas NETs (all < 5% incidence) [6]. However, in cases of metastatic disease, appendiceal NETs had the highest rate of peritoneal involvement at about 70% [6].

**Table 1:** Literature review of the incidence of PM in SBNET

Authors	Study Type	Study Years	Patient Population (PM incidence)
Madani et al. *Ann Surg Oncol* 2017 [6]	Retrospective Analysis - Multi-Institutional National Registry	2007–2013	4114 patients with GEPNETs (13% of patients with SBNET had PM)
Das et al. *J Med Surg Pathol*. 2019 [67]	Retrospective analysis - Single Institution	1994–2015	208 SBNET patients (64 patients with PM − 30.8%)
Wright et al. *Am J Surg Pathol* 2020 [9]	Retrospective analysis - Single Institution	1994–2017	219 SBNET patients (67 patients with PM − 30.6%)
Wonn et al. *Surgery* 2021 [4]	Prospective Analysis - Single Institution	2008–2019	323 patients with SBNET (98 with PM − 30%)
Tsoli et al. *J Endocrinol Invest* 2024 [68]	Retrospective Analysis - Multi-Institution	1999–2020	1777 NETs patients (183 or 10.3% incidence of PM and most were SB primary)
Chang et al. Ann Surg Oncol 2025 [21]	Retrospective analysis - Single Institution	2005–2024	396 patients with SBNET (119 with PM − 30.1%)

## Diagnosis and Surveillance of PM in SBNET

Peritoneal disease may be difficult to diagnose preoperatively, especially due to the significant heterogeneity in clinical symptoms and limitations of imaging modalities in detecting relatively low burden of PM. Symptoms of PM are dependent on the extent and location of peritoneal disease. Occult PM are often asymptomatic or may present with vague, nonspecific symptoms. Common PM symptoms include abdominal bloating or distension, abdominal pain, weight loss, change in bowel movements or change in appetite. PM nodules implanted on the bowel wall can cause mechanical obstruction by narrowing or kinking the bowel. As PM disease worsens, there may be mass effect from growing lesions, which may ultimately lead to complications including organ dysfunction. Larger lesions may narrow blood vessels or cause ureteral obstruction [19, 20]. On physical exam, while there may not be any appreciable signs, potential findings include abdominal distension, tenderness, palpable masses, fluid wave or dullness to percussion. Neuroendocrine tumor marker levels (which may include serotonin, chromogranin A, neurokinin A, and pancreastatin) can suggest overall tumor burden and preoperative assessment provides a baseline for future comparison. Patients with higher T stage, female sex, elevated pancreastatin levels, and synchronous liver metastases have higher risk for developing PM [21]. 

Imaging evaluation is critical in the preoperative diagnosis of PM. In patients presenting with abdominal distension, abdominal ultrasound may identify the presence of ascites and omental and mesenteric thickening or abdominal masses [22, 23]. More typically, computerized tomography (CT) scans are used. CT imaging findings of PM include ascites, omental or mesenteric infiltration/thickening or scattered peritoneal nodules [24]. The sensitivity and specificity of CT imaging for identifying peritoneal nodules >1 cm are 85% and 86%, respectively [25, 26]. However, with lesions < 1 cm in size, the sensitivity decreases significantly to 7–28%.

For neuroendocrine malignancies, somatostatin receptor (SSTR) DOTA-PET/CT imaging is another powerful diagnostic tool [27]. These imaging modalities depends upon high expression of SSTRs in NETs. This functional imaging was initially performed with ^111^In-pentetreotide scintigraphy (OctreoScan) [28]. More recently, ^68^Ga-DOTATATE, ^68^Ga-DOTATOC, ^68^Ga-DOTANOC, and ^64^Cu-DOTATATE (collectively DOTA PET/CT) have replaced OctreoScan due to improved sensitivity and specificity [29–33]. In our practice, we routinely utilize DOTA PET/CT preoperatively for diagnosis, staging, and identification of distant metastases. DOTA PET/CT can identify bone, peritoneal and lymph node metastases with improved efficacy over MRI or CT scans [28]. 

Some patients will develop PM in the postoperative surveillance period. In our study of 396 patients with SBNET over 20 years, we found that ~ 5% of patients without PM will develop PM after initial surgical resection with a median time to PM diagnosis of 54 months. This highlights the importance of long term follow up of these patients [34]. For patients with liver metastases who undergo liver cytoreduction at the time of initial oncologic resection, we perform a CT scan 3 months postoperatively then every 6 months for the first 2 years, extending out to 1 year after that if there are no metastases. We do not routinely utilize DOTA PET/CT imaging for surveillance but obtain these scans when there are clinical, biochemical or imaging signs concerning for progression. This is when treatment with peptide receptor radiotherapy (PRRT) will be considered, and it is an important time to re-evaluate the presence of SSTRs and extent of disease [35, 36]. 

## Disease Distribution and Surgical Management

**Fig. 1:**
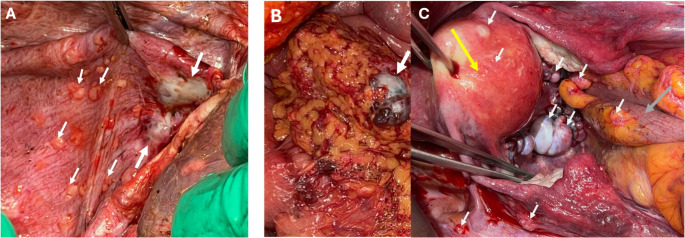
Intraoperative Photos of Peritoneal Metastases. (**A**) Metastases to the diaphragm (white arrows) of varying sizes. The larger 2 required full thickness excision and primary diaphragmatic repair. (**B**) Omental Metastases (white arrows) with one large lesion and multiple smaller lesions, treated by omentectomy. (**C**) “Drop Metastases” to the pelvis, involving the side wall, uterus, ovaries, posterior vagina, and rectosigmoid colon. The uterus (yellow arrow) is retracted anteriorly and rectosigmoid colon (grey arrow) pulled superiorly. Ureteral stents were placed, allowing for safer tumor excision requiring posterior vaginectomy and imbrication of rectal wall (rather than low anterior resection); oophorectomy was also performed, and removal of all other > 5 mm lesions with argon beam coagulation of lesions < 5 mm

Classification of the degree of PM in SBNET can be done using the Lyon prognostic index, as first recommended by ENETS guidelines [8]. The Lyon Score (LS) is a 0–4 classification incorporating extent of lesions (i.e. localized vs. disseminated) and lesion size [8]. A Lyon score of 0 corresponds to no macroscopic disease, 1 to PM less than 5 mm localized in one part of the abdomen, 2 to PM less than 5 mm diffusely throughout the whole abdomen, 3 to PM 5 mm to 2 cm and 4 to PM >2 cm in size. The most common region for peritoneal disease involvement is the pelvis, where “drop metastases” form, which may be found in the cul-de-sac, or on the rectosigmoid colon, bladder, and ovaries (Fig. 1A). Additional frequent sites of PM are the diaphragm, lateral peritoneal walls, omentum, and intestinal mesentery. Completeness of operative resection should be weighed against the patient’s risk of morbidity and mortality. Current NANETS guidelines for PM recommend “removal of as much disease as possible while minimizing risks” [37]. Recently, we have shown that in SBNET patients that undergo surgery, those who have cytoreduction of PM have higher rates of major complications (i.e. Clavien Dindo 3 or 4), specifically, increased risk of organ space surgical site infection (SSI) and postoperative bowel obstruction [21]. These risks should be explained to patients undergoing these procedures. While complete cytoreduction to a LS of 0 is ideal, cytoreduction of PM to an LS of 1 does confer an oncologic survival benefit. In Wonn et al., the median survival time for patients with a closing LS *≥* 3 compared to LS *≤* 1 was 39 months compared to 76 months (*p* = 0.04) when liver metastases were cytoreduced >70% (Table 2) [4].

Tumor biology should impact decision making. For NETs, tumor grade, as determined by Ki-67 (%), a marker of cellular proliferation, is helpful in predicting cancer behavior and prognosis. Neuroendocrine carcinomas (NECs) are poorly differentiated and high grade tumors. NECs of the small bowel are rare and not discussed in the scope of this review, and are associated with poor survival. For grade 1 or 2 NETs with PM, upfront surgery should be considered if near complete cytoreduction is possible. For grade 3 NETs with PM, the management is more nuanced regarding the sequence of systemic or surgical treatments [38–40]. Clinical assessment of disease biology, molecular profiling and multi-disciplinary discussions are key for therapeutic decision making among these patients [41]. 

In general, an aggressive approach towards cytoreduction of PM associated with SBNETs is encouraged, with a goal of achieving a completeness of cytoreduction (CC) score of 0 or 1 (i.e. no visible tumor remains or residual tumor nodules ≤ 2.5 mm). The strategy depends on location, nodule size and density of disease. In general, lesions larger than 5–10 mm should be removed (i.e. mesenteric implants, diaphragm, pelvic or peritoneal side wall) while lesions less than 5 mm may be ablated with argon beam or other means. For larger lesions or if there is significant nodularity such as omental (Fig. 1B) or diaphragmatic caking, more extensive resections may be required. Smaller diaphragm lesions may be excised by just peeling them off the muscle, obviating the need for thoracic drainage. When larger lesions caking the diaphragm are excised, primary repair or with placement of Gore-tex mesh for larger defects may be needed, with short term pleural drainage. Formal peritoneal stripping may not be necessary if local excision of larger nodules can be achieved. The peritoneal surfaces of intraabdominal and pelvic organs may be involved. Lesions on the bowel wall may be shaved off and the serosa reinforced with Lembert sutures. If the rectosigmoid is significantly narrowed by tumor, then low anterior resection should be considered. If ureters are involved, resection and primary re-anastomosis may be needed. When the ovaries are involved (Fig. 1C), oophorectomy is recommended.

In 2025, the Peritoneal Surface Malignancy Consortium published consensus guidelines for the management of PM from NETs as an update to the prior 2018 Chicago Consensus guidelines [42, 43]. They outline the importance of understanding goals of surgery. For surgically treatable peritoneal metastases in G1 or G2 NETs, cytoreduction is recommended. If there are liver metastases, they should be approached surgically concurrently if one is able to cytoreduce >70%. If the extent of disease is too great or patient comorbidities are prohibitive to an aggressive cytoreductive attempt, palliative surgery may be considered to remove bulky disease which can cause bowel obstruction [44]. The guidelines highlight that these surgeries are demanding and can be technically challenging and when possible, these patients should be referred to high volume clinical centers.

## Impact of Peritoneal Metastases on Prognosis and Morbidity

In general, PM is a poor prognostic factor, yet its independent contribution to survival in NETs remains under investigation. As in other more aggressive tumor types such as ovarian or colorectal cancer, NET PM are often accompanied by metastases to other areas, which may be more rapidly progressive and a greater threat to the patient. A retrospective analysis by Norlen et al., which included 603 patients with SBNETs from 1985 to 2010, found that patients with PM have significantly worse 5-year OS compared to those without PM (52% vs. 79%, *p* < 0.01) [28]. Yet in this analysis, only 67% of patients had distant metastatic disease. PM is infrequently an isolated event, and often occurs in the setting of other local and distant metastatic disease, making the impact of peritoneal versus other synchronous metastases difficult to assess.

Another question is whether in the setting of metastatic disease PM conveys worse prognosis than metastasis to other sites. Gudmundsdottir et al. found that there was no difference in OS between patients with liver metastases only and liver metastases plus PM when complete cytoreduction was able to be performed, with a median OS of approximately 11 years [5]. Patients with incomplete cytoreduction of liver or PM disease (defined by < 90% disease debulking and closing Lyon score of 1 or 2) had worse outcomes than those who had all disease removed (median OS of 7.1 years vs. 11.2 years). However, there was no difference in outcomes within the incompletely cytoreduced subgroup with isolated liver metastases vs. liver metastases + PM. These data agree with previous studies by Benhaim et al. and Wonn et al. [4, 45] where it was suggested that greater disease burden may lead to worse outcomes, not the presence of PM.

## Role of Hyperthermic Intraperitoneal Chemotherapy for Peritoneal Metastases

Cytoreductive surgery (CRS) with Hyperthermic Intraperitoneal Chemotherapy (HIPEC) has been used for pseudomyxoma peritonei, appendiceal neoplasms, colorectal, gastric, and other cancers [46]. In ovarian cancer requiring neoadjuvant chemotherapy, addition of hyperthermic intraperitoneal chemotherapy is associated with significant survival benefit [47]. Cytoreduction, even if complete, only treats visible disease and thus additional locoregional methods have been suggested to treat microscopic disease. HIPEC involves the delivery of chemotherapy in a heated environment (which augments uptake and sensitivity to chemotherapy) to help eliminate these microscopic tumor cells. Multiple chemotherapy regimens exist. The depth of penetration of the heated chemotherapy is approximately 2–3 mm, thus a relatively complete cytoreduction is required for HIPEC to be effective. There currently exist no randomized trials investigating HIPEC in NETs and only a few retrospective series (Table 2). The series by Elias et al. retrospectively analyzed 41 patients with NET-derived PM; 28 were treated with CRS + HIPEC and 13 had CRS alone. No difference in overall survival was found between groups and the recurrence rate of PM was 47%. The HIPEC protocol used in this study included intravenous (IV) infusion of 20 mg/m^2^ leucovorin and 400 mg/m^2^ 5-fluorouracil, followed by HIPEC, performed over 30 min at 43 degrees Celsius with oxaliplatin alone or mixed with irinotecan in 2L/m^2^ of 5% Dextrose [48]. Another French study with 67 patients who had CRS for PM from SBNET included 36 patients receiving HIPEC and 31 having CRS only. No survival benefit was found by adding HIPEC (5-year OS 92 vs. 75% in CRS-only and CRS/HIPEC groups, *p* = 0.3). However, patients in the CRS/HIPEC group had significantly higher disease burden and greater grade III/IV postoperative morbidity compared to those receiving CRS alone (50% vs. 3.4%, *p* < 0.01) [49]. Based on these data, the updated peritoneal surface malignancy consortium 2025 consensus guidelines do not recommend HIPEC for PM in NETs [43]. In summary, there are no recommendations in favor of using HIPEC or other intraperitoneal therapies for PM from SBNET.

## Systemic Therapies for Peritoneal Metastases

Somatostatin analogs (SSAs) are frequently first line treatment for metastatic SBNETs, the majority of which express somatostatin receptors. SSAs, in addition to aiding in symptom control (which is related to tumor hormone hypersecretion), may exert antiproliferative effects on NETs. This idea comes from the PROMID and CLARINET trials which demonstrated improved progression free survival (PFS) with SSAs, Octreotide LAR and Lanreotide, respectively, as compared to placebo [50, 51]. Chemotherapy and biologic agents may have a role in NETs progressing with SSA therapy. Everolimus, an mTOR inhibitor, has been found to have some effect in improving PFS in advanced lung and gastrointestinal NETs [52]. However, extrapolation of this result to PM is difficult due to the small numbers of patients with PM in the study. The RADIANT-4 phase 3 trial of Everolimus included only 25 (12%) patients with PM in the Everolimus arm and 8 (8%) patients with PM in the placebo arm. No subgroup analyses in these populations were performed. There are limited data supporting the use of cytotoxic chemotherapies in certain patient populations. Capecitabine/temozolomide (CAPTEM) while more studied in pancreatic NETs, has shown some activity in high-grade small bowel NETs, however no randomized controlled trials exist to support this [53]. The CABINET (phase 3 randomized, placebo-controlled) trial found cabozantinib (60 mg daily dosing), a tyrosine kinase inhibitor, to improve PFS over placebo for pancreatic and extra-pancreatic NETs with previous progression on prior systemic therapy [54]. In the extra-pancreatic NET group of the CABINET trial, the majority of patients had low G1/2 disease and there were no data regarding rate of PM. Historically, the difficulty of detecting peritoneal metastases radiographically and measuring their response to treatment has limited inclusion of these patients in trials for advanced GI malignancies, and this is likely true for NETs as well [55]. As the utility and efficacy of these systemic treatments can be nuanced, clinical decision making for systemic therapy in PM should be undertaken within a multidisciplinary care team.

### Effect and Role of PRRT for Peritoneal Metastases

One of the most significant improvements in the treatment of advanced or unresectable NETs has been the development and approval of peptide receptor radionuclide therapy (PRRT). Based on promising results of the pivotal phase III NETTER-1 trial and a large single-center analysis on the safety and efficacy of PRRT in GEPNETs and bronchial NETs from the Netherlands, the US Food and Drug Administration (USFDA) approved ^177^Lu-DOTATATE (or Lutathera^®^) for advanced SSTR-avid GEP-NETs in January 2018 [56, 57]. NETTER-1 compared ^177^Lu-DOTATATE plus long-acting octreotide 30 mg/q 28 days with high dose octreotide 60 mg for patients who had disease progression on fixed dose octreotide. In the NETTER-1 trial, only a minority of patients had peritoneal disease (*n* = 17, 15%). Of these, 7 (6%) were in the ^177^Lu-DOTATATE arm. The subsequent NETTER-2 trial compared ^177^Lu-DOTATATE plus octreotide 30 mg/q 28 days against high-dose long acting octreotide (60 mg monthly) as first-line systemic therapy in patients with higher-risk, well-differentiated grade 2 NETs (Ki-67 ≥ 10–20%) and grade 3 NETs (Ki-67 >20% to ≤ 55%) [58]. A relatively higher percentage of patients with peritoneal metastases were accrued to the PRRT arm (17%), however outcomes were not separately reported for this subgroup.

Although the introduction of PRRT has allowed for significant improvement in outcomes in NET patients, retrospective data suggests that PRRT in patients with peritoneal disease is associated with significant risk of complications, such as bowel obstruction. NETTER-1 recorded only 2 hospitalizations for small bowel obstruction, which likely reflects the low incidence of peritoneal metastases among patients included in the study [56]. In NETTER-2, small bowel obstruction was noted in 3.4% patients, all of whom were in the PRRT arm [58]. In a retrospective analysis of 135 patients with GEPNETs (the majority were G1/2 and of small bowel primary), treatment with ^177^Lu or ^90^Y90-SSTR-directed PRRT was associated with peritoneal disease progression in 37.5% and bowel obstruction or ascites in 28.1% of cases [59]. A retrospective analysis included 20 patients with peritoneal carcinomatosis or mesenteric fibrosis that underwent treatment with 2 cycles of PRRT reported that 15% of patients developed small bowel obstruction within 3 months of PRRT [60]. Small bowel obstruction after PRRT is thought to result from radiation-induced inflammation or radiogenic edema [61]. High-dose corticosteroids have been shown to be modestly helpful in temporarily mitigating the risk of bowel obstruction associated with PRRT in these patients [62]. 

Pre-clinical data highlight that the tumor microenvironment of peritoneal/mesenteric NET metastases are highly fibrotic and hypoxic. This is largely due to the secretion of serotonin, TGF-β and other fibrogenic substances from these metastatic sites, which ultimately recruit fibroblasts and remodel the extracellular matrix, resulting in a desmoplastic reaction [63]. This not only impedes drug penetration but also induces local hypoxia. PRRT using alpha emitters such as ^225^Ac or ^212^Pb are currently under active investigation and carry the potential to be more cytotoxic to tumors than beta PRRT, such as ^177^Lu-DOTATATE [64]. Alpha emitters cause double-stranded DNA breaks, and may be less dependent on oxygen in the tumor microenvironment. This may allow alpha PRRT to overcome hypoxia-associated radioresistance in fibrotic niches such as peritoneal carcinomatosis with mesenteric fibrosis [65]. The therapeutic efficacy and safety of alpha PRRT remains to be determined and clinical trials are ongoing including ALPHAMEDIX02 (NCT05153772) using ^212^Pb-DOTAMTATE, ACTION-1((NCT05477576) using RYZ101 (^225^Ac-DOTATATE) and VMT-alpha-NET (NCT06148636) using [^212^Pb]VMT-alpha-NET.

## Palliative Therapy for PM in SBNET Patients

Challenging decisions exist in managing patients with advanced peritoneal disease too extensive for adequate cytoreduction. It is reasonable to make every attempt to cytoreduce patients at the time of presentation, but a more difficult question is when and how often to go back to try to treat symptomatic recurrences surgically. Patients may have repeated episodes of small bowel obstruction over time, some of which may be improved with surgical treatment. However, repeat explorations usually require extensive adhesiolysis of matted loops of bowel, and the number of peritoneal lesions may be daunting. Exploration and adhesiolysis risk enterotomoties, which even if repaired can lead to enterocutaneous fistulas, markedly reducing quality of life. It is also common to need to resect a substantial length of bowel involved with the peritoneal nodules, which may lead to short gut syndrome. If there is rectosigmoid obstruction, one must decide whether to do low anterior resection with pelvic anastomosis versus a diverting colostomy. The former may be better for quality of life, but there is risk of leak, while the latter may be hard for patients to accept, but can still significantly improve quality of life.

Decision making is complex, and it is important to take into account the goals of the patient and the risks of surgery. Other important factors in deciding whether to operate include the burden of disease by DOTA-PET and CT, and whether the surgeon thinks they can effectively cytoreduce lesions causing recurrent problems. The presence of peritoneal disease alone on imaging may not be worth another exploration unless the patient is experiencing significant symptoms. The nutritional status of the patient, their co-morbidities, the grade of tumor, and their functional status are also important determinants. Multi-disciplinary input is important to consider non-surgical options, as well as invoving the palliative care team. If the patient is considered unlikely to benefit from repeat cytoreductive surgery, then consideration to placing long term venous access for either IV fluids or parenteral nutrition should be considered. For end-stage patients with recurrent small bowel obstructions, placing a venting gastrostomy tube may be extremely helpful to allow for intermittent decompression so patients do not need to vomit. High-dose H2-blockers can reduce the volume of gastric secretions to palliate symptoms, and a short course of steroids helps obstructive symptoms by reducing peri-tumoral inflammation [66]. Occasionally patients may be able to tolerate tube feeds, so a jejunostomy tube can also be considered, or a double lumen gastro-jejunostomy tube for both venting and feeds.

## Conclusions

PM occur in up to 30% of SBNET patients and are associated with worse prognosis and increased disease morbidity. Appropriate workup and imaging are critical in preoperative planning. Many patients with PM will have disease recurrence and some without PM may develop PM over their disease course, thus interval imaging is necessary for surveillance. While surgery is the mainstay of management, there are additional therapeutic modalities that can be considered in a carefully selected patient population after multi-disciplinary discussion. Given the high prevalence of PM in this disease, future trials should seek to include these patients and evaluate efficacy in this population. Despite the increased risks conferred by PM, an aggressive approach to surgical cytoreduction of disease improves long-term survival, and the presence of PM along with synchronous liver metastases should not preclude operative resection.

**Table 2:** Literature review of operative interventions for PM in SBNET

Authors	Study Type	Patient population (PM rates)	Intervention Investigated	Results	Conclusions
Wonn et al. *Surgery* 2021	Prospective Analysis	323 patients with SBNET (98 with PM − 30%)	CRS for PM in the setting of liver metastases	Median OS − 67 months Closing Lyon score = 0–135 months Closing Lyon score *≤* 1–76 months Closing Lyon score *≥* 3–32 months *All Patients with > 70% liver disease cytoreduced	Cytoreduction of PM should be performed in conjunction with liver cytoreduction. Best outcomes are with a complete cytoreduction but there is a survival benefit with cytoreduction to Lyon Score *≤* 1.
Gudmundsdottir et al. *Ann Surg Oncol* 2024	Prospective Analysis	261 SBNET patients with liver metastases (50 with PM)	CRS for PM in the setting of liver metastases	Patients with complete cytoreduction of mets - isolated liver metastases vs. both liver and peritoneal metastases (11.5 vs. 11.2 years, *p* = 0.10). Patients with incomplete cytoreduction of mets (< 90% debulking of liver metastases and closing Lyon score of 1/2) - isolated liver metastases vs. both liver and peritoneal metastases (6.4 vs. 7.1 years, *p* = 0.12).	Patients with metastatic SBNET to liver and peritoneum can have good long term survival with aggressive cytoreduction of both
Elias et al. *Surgery* 2014	Prospective comparative study	41 patients with NET derived PM (31 (76%) from SB primary) − 28 with CRS + HIPEC and 13 with CRS alone)	CRS with or without HIPEC	5 year OS of 69%. No difference in 2 year OS between CRS alone and CRS + HIPEC groups (73% vs. 88%, *p* = 0.73). No difference in 2 year peritoneal RFS between CRS alone and CRS + HIPEC groups (67% vs. 68%, *p* = 0.87).	There is no data to suggest benefit of HIPEC in addition to CRS.
Hajjar et al. *Eur J Surg Oncol* 2022	Retrospective analysis of prospective database	67 patients with PM from SBNET (36 with CRS + HIPEC and 31 with CRS alone)	CRS with or without HIPEC	No difference in 5 year OS between CRS alone and CRS + HIPEC groups (91.6% vs. 74.5%, *p* = 0.32). No difference in 5 year PFS between CRS alone and CRS + HIPEC groups (0% vs. 30.8%, *p* = 0.78). There was significantly increased rates of Grade III-IV adverse events in CRS + HIPEC group (50% vs. 3.4%, *p* < 0.01).	There is no data to suggest benefit of HIPEC in addition to CRS. There is increased morbidity risk.
Chang et al. *Ann Surg Oncol* 2025	Retrospective analysis of prospective database	396 patients with SBNET (119 with PM − 30.1%). All patients received operative intervention (primary and/or metastases)	CRS	Higher T stage, female sex, presence of liver metastases and elevation in pancreastatin are associated with increased risk of PM. PM not an independent risk factor for OS or RFS. Median OS of PM patients: 109 months	Operative management of PM is associated with increased morbidity, however aggressive management of PM in SBNET can lead to long-term survival.

*SBNET small bowel neuroendocrine tumor, *CRS* cytoreductive surgery, *OS* overall survival, *PM* peritoneal metastases, *HIPEC* hyperthermic

## Key References

- Wonn SM, Limbach KE, Pommier SJ, Ratzlaff AN, Leon EJ, McCully BH, et al. Outcomes of cytoreductive operations for peritoneal carcinomatosis with or without liver cytoreduction in patients with small bowel neuroendocrine tumors. Surgery. 2021;169(1):168-74. 10.1016/j.surg.2020.03.030.
  - This study demonstrates that aggressive cytoreduction of PM is frequently possible and should occur even in the setting of liver metastases with best outcomes occur from adequate cytoreduction of both (liver to >70% and PM to closing Lyon Score of <1).
- Strosberg JR, Al-Toubah T, Pelle E, Smith J, Haider M, Hutchinson T, et al. Risk of Bowel Obstruction in Patients with Mesenteric or Peritoneal Disease Receiving Peptide Receptor Radionuclide Therapy. J Nucl Med. 2021;62(1):69-72
  - This study published a few years after the NETTER-1 trial highlights the real risk of bowel obstruction in patients with PM who receive PRRT.

## Data Availability

No datasets were generated or analysed during the current study.
